# EGFR and MUC1 as dual-TAA drug targets for lung cancer and colorectal cancer

**DOI:** 10.3389/fonc.2024.1433033

**Published:** 2024-11-27

**Authors:** Huilin Cui, Qianqian Yu, Qumiao Xu, Chen Lin, Long Zhang, Wei Ye, Yifei Yang, Sijia Tian, Yilu Zhou, Runzhe Sun, Yongsheng Meng, Ningning Yao, Haizhen Wang, Feilin Cao, Meilin Liu, Jinfeng Ma, Cheng Liao, Ruifang Sun

**Affiliations:** ^1^ Department of Histology and Embryology, Shanxi Medical University, Taiyuan, Shanxi, China; ^2^ Department of Tumor Biobank, Shanxi Province Cancer Hospital/Shanxi Hospital Affiliated to Cancer Hospital, Chinese Academy of Medical Sciences/Cancer Hospital Affiliated to Shanxi Medical University, Taiyuan, China; ^3^ Department of Translational Medicine, Shanghai Shengdi Medicine Co. Ltd., Shanghai, China; ^4^ School of Basic Medicine, Shanxi Medical University, Jinzhong, China; ^5^ Department of Radiobiology, Shanxi Province Cancer Hospital/Shanxi Hospital Affiliated to Cancer Hospital, Chinese Academy of Medical Sciences/Cancer Hospital Affiliated to Shanxi Medical University, Taiyuan, China; ^6^ Department of Hepatobiliary and Pancreatogastric Surgery, Shanxi Province Cancer Hospital/Shanxi Hospital Affiliated to Cancer Hospital, Chinese Academy of Medical Sciences/Cancer Hospital Affiliated to Shanxi Medical University, Taiyuan, China

**Keywords:** EGFR, MUC1, TAA, LUAD, CRC

## Abstract

**Background:**

Epidermal growth factor receptor (EGFR) is a key protein in cellular signaling that is overexpressed in many human cancers, making it a compelling therapeutic target. On-target severe skin toxicity has limited its clinical application. Dual-targeting therapy represents a novel approach to overcome the challenges of EGFR-targeted therapies.

**Methods:**

A single-cell tumor-normal RNA transcriptomic meta-atlas of lung adenocarcinoma (LUAD) and normal lung tissues was constructed from published data. Tumor associated antigens (TAAs) were screened from the genes which were expressed on cell surface and could distinguish cancer cells from normal cells. Expression of MUC1 and EGFR in tumors and normal tissues was detected by immunohistochemistry (IHC), bulk transcriptomic and single-cell transcriptomic analyses. RNA cut-off values were calculated using paired analysis of RNA sequencing and IHC in patient-derived tumor xenograft samples. They were used to estimate the abundance of EGFR- and MUC-positive subjects in The Cancer Genome Atlas Program (TCGA) database. Survival analysis of EGFR and MUC1 expression was carried out using the transcription and clinical data from TCGA.

**Results:**

A candidate TAA target, transmembrane glycoprotein mucin 1 (MUC1), showed strong expression in cancer cells and low expression in normal cells. Single-cell analysis suggested EGFR and MUC1 together had better tumor specificity than the combination of EGFR with other drug targets. IHC data confirmed that EGFR and MUC1 were highly expressed on LUAD and colorectal cancer (CRC) clinical samples but not on various normal tissues. Notably, co-expression of EGFR and MUC1 was observed in 98.4% (n=64) of patients with LUAD and in 91.6% (n=83) of patients with CRC. It was estimated that EGFR and MUC1 were expressed in 97.5% of LUAD samples in the TCGA dataset. Besides, high expression of EGFR and MUC1 was significantly associated with poor prognosis of LUAD and CRC patients.

**Conclusions:**

Single-cell RNA, bulk RNA and IHC data demonstrated the high expression levels and co-expression patterns of EGFR and MUC1 in tumors but not normal tissues. Therefore, it is a promising TAA combination for therapeutic targeting which could enhance on-tumor efficacy while reducing off-tumor toxicity.

## Introduction

Epidermal growth factor receptor (EGFR), a tyrosine kinase receptor whose activation leads to receptor dimerization and tyrosine autophosphorylation, mediates tumor cell survival and proliferation in lung cancer, colorectal cancer (CRC) and breast cancer (1–4). Monoclonal antibody inhibitors of EGFR have been developed for cancer therapy in the last two decades, including cetuximab and necitumumab approved by the U.S. Food and Drug Administration (FDA) for lung adenocarcinoma (LUAD) and squamous cell lung cancer (5, 6), and panitumumab and cetuximab approved by the FDA and European Medicines Agency (EMA) for the treatment of metastatic CRC (7). However, anti-EGFR therapy can be marred by chronic and disfiguring adverse reactions such as acne-like rash, abnormal hair growth, and ocular abnormalities, which could worsen life qualities of patients and even trigger treatment termination (7).

Tumor associated antigens (TAAs) are important tumor targets for drug development with abnormal expression on tumor cells (8). While drugs targeting single TAA faced obstacles such as on-target, off-tumor toxicity and antigen escape, strategies targeting dual TAAs could improve the selectivity of tumor cells and reduce drug toxicity (9), through stronger binding to tumor cells expressing dual TAAs rather than normal tissues expressing a single target. To find an optimal TAA target to combine with EGFR, we constructed a computational pipeline which integrated multiple single-cell RNA sequencing datasets of both tumor and normal tissues from public sources. From a pool of TAAs identified by single-cell analysis, transmembrane glycoprotein mucin 1 (MUC1) was distinguished as a candidate target with promising druggability, as affirmed by extensive literature review and evaluation of its therapeutic tractability.

Through analysis of an independent lung cancer single-cell dataset, we showed that co-expression of EGFR and MUC1 was specific to tumor cells in the tumor microenvironment. We then confirmed that the expression patterns of EGFR and MUC1 in tumor tissues by immunohistochemistry (IHC). EGFR and MUC1 were co-expressed in 63 (98.4%) of the 64 patients with LUAD and in 76 (91.6%) of the 83 patients with CRC. In addition, we performed paired analysis of RNA sequencing and IHC data of patient-derived tumor xenograft (PDX) samples, and estimated that EGFR and MUC1 were expressed in 97.5% of LUAD samples of The Cancer Genome Atlas Program (TCGA) database. High expression of EGFR and MUC1 was associated with poor prognosis in LUAD and CRC. Together, our results illustrated the expression patterns of EGFR and MUC1 in tumor and normal tissues, and suggested that they were promising drug targets for developing cancer therapies targeting dual TAAs.

## Materials and methods

### Single-cell RNA sequencing data processing

For LUAD, a tumor-normal single cell meta-atlas was constructed through integrating single-cell RNA data of primary LUAD samples and normal lung tissue samples from public single cell datasets (Kim et al. (10), Qian et al. (11), E-Madissoon et al. (12), Braga et al. (13), **Supplementary Table 1**). An external single cell dataset from Wu et al. (14) was used for validation (**Supplementary Table 1**). Files of raw UMI count matrices were downloaded from GEO (15), Array Express (16) or author-referred websites and imported into Scanpy (17). To ensure data quality, cells with fewer than 3 detected genes, fewer than 200 unique molecular identifier (UMI) counts, or mitochondrial gene percentages greater than 20% were filtered out.

Next, a global-scaling normalization method was applied, adjusting the read count in each cell to 10^6 and subsequently performing log-transformation. The top 2000 highly variable genes (HVG) were identified and used in the principal component analysis (PCA), from which the top 30 components were retained for downstream dimensional reduction and clustering analysis using default parameters.

Due to the datasets being collected from different studies, the dataset ID was considered as the batch variable and corrected using a ridge-regularized linear regression model. Additionally, the BBKNN algorithm with default parameters was applied for evaluation and visualization (18).

For cell type annotation, a two-step process was employed. First, SingleR (19) was used for automatic cell annotation with HumanPrimaryCellAtlasData as the reference. Following this, curated markers or available annotation files from previous research (20) were manually applied to refine the cell type annotations based on the SingleR predictions (marker genes were listed in the **Supplementary Table 2**). This resulted in the construction of a tumor-normal single-cell meta-atlas for the discovery of TAAs.

### Malignant cell identification

The raw count matrices of tumor samples with manually annotated cell labels were imported into the CopyCat (21) analysis with default parameters. Immune cells and fibroblasts from each sample served as normal cell references. Epithelial cells with diploid or undefined predictions were excluded from further analysis. In contrast, epithelial cells with aneuploid predictions were identified as malignant cells and retained in the filtered tumor-normal single-cell meta-atlas.

### Random-forest construction

The filtered tumor-normal single-cell meta-atlas was randomly divided into training, testing, and validation datasets at 6:2:2 ratio. A random forest classifier with 1000 trees was built using the R package ‘randomforest’ (22). The model was initially trained on training datasets with 10-fold cross-validation and then evaluated on testing datasets to assess performance. The average AUC score was calculated using the ROCR R package (23) to select the best fitting model. Feature importance scores were calculated to determine the influence of genes in the final random forest model. The highest-ranked genes by MeanDecreaseAccuracy were overlapped with cell surface proteins from the in silico human surfaceome database (24) to extract the top 100 genes with surface expression.

### Expressing cell fraction

The raw counts of each gene in the filtered tumor-normal single-cell meta-atlas were transformed into a binary classification to illustrate the expression pattern. A raw count greater than 0 was considered an expressed pattern (True), while a raw count of 0 was considered a non-expressed pattern (False). The ECF of a gene was calculated as the percentage of cells with an expressed pattern in a specific cell group.

### LUAD, CRC, breast cancer tissues and normal samples

Paraffin sections of normal tissue samples for IHC experiments were sourced from Guilin Fanpu Biotech, Inc. with ethical approval for research, and the sample characteristics were provided in **Table 1**. Paraffin sections and tissue microarrays (TMAs) of human LUAD, CRC and breast cancer tissues from randomly and anonymously selected patients (LUAD, n=64; CRC, n=83; breast cancer, n=20) were provided by Shanxi Province Cancer Hospital with the patients’ informed consent. Patient characteristics, including age, sex, stage, tumor size, etc., were listed in **Table 2**. The pathological subtype of LUAD diagnosis was made in accordance with the 2015 World Health Organization Classification (25). The TMAs of LUAD and CRC PDX models were also obtained from Shanxi Province Cancer Hospital.

**Table 1:** Characteristics of normal tissue samples.

Sample ID	Donor Sex	Donor Age	Tissue origin	Tissue condition
F17-0863	Female	48	Breast	Normal adjacent tissue of breast tumor
F17-1600	Female	27	Placenta	Normal
F17-1649	Female	8	Tonsil	Normal
F19-0082	Male	42	Adrenal gland	Adrenocortical hyperplasia
F17-4595	Male	82	Prostate	Benign prostatic hyperplasia
F20-1535	Female	69	Lung	Normal adjacent tissue of lung tumor
F20-1596	Male	48	Liver	Normal adjacent tissue of hepatic inflammatory pseudotumor
F19-1687	Male	51	Kidney	Kidney rupture
F19-0211	Male	21	Stomach	Normal stomach tissue from a donor with peptic ulcer
F18-4236	Female	48	Ovary	Ovarian tissue from a donor with uterine leiomyoma
F17-2257	Male	54	Small intestine	Normal intestinal tissue from a donor with intestinal obstruction
F18-0991	Male	72	Testicles	Normal
F20-1289	Male	55	Esophagus	Normal adjacent tissue of esophageal tumor
F20-0956	Male	52	Pancreas	Pancreas tissue from a donor with ampullary adenocarcinoma
F20-1423	Male	34	Colon	Normal adjacent tissue of colonic lipoma
F20-0646	Female	45	Skin	Breast skin tissue

**Table 2:** Characteristics of LUAD, CRC and BRCA patients with tissue samples analyzed in IHC.

Cancer type	LUAD	CRC	BRCA
Characteristics, n (%)	n = 64	n = 83	n=20
Sex			
Male	34 (53.1%)	54 (65.1%)	0 (0.0%)
Female	30 (46.9%)	29 (34.9%)	20 (100.0%)
Age(years)			
<65	48 (75.0%)	51 (61.4%)	16 (80.0%)
≥65	16 (25.0%)	32 (38.6%)	4 (20.0%)
Tumor size			
≤5 cm	62 (96.9%)	25 (30.1%)	19 (95.0%)
>5 cm	2 (3.1%)	56 (67.5%)	1 (5.0%)
grading			
I	3 (4.7%)	4 (4.8%)	0 (0.0%)
I-II	5 (7.8%)	27 (32.5%)	0 (0.0%)
II	24 (37.5%)	26 (31.3%)	14 (70.0%)
II-III	6 (9.4%)	9 (10.8%)	0 (0.0%)
III	12(18.8%)	6 (7.3%)	1 (5.0%)
Lymphovascular invasion			
negative	40 (62.5%)	53 (63.9%)	7 (35.0%)
positive	24 (37.5%)	28 (33.7%)	13 (65.0%)
AJCC Stage			
I	39 (60.9%)	22 (26.5%)	7 (35.0%)
II	1 (1.6%)	31 (37.3%)	0 (0.0%)
III	24 (37.5%)	28 (33.7%)	13 (65.0%)

### IHC

The IHC protocol for MUC1 and EGFR was as follows:

i) For antigen retrieval, sections were treated with retrieval solution (high pH) in Leica BOND-MAX for both EGFR and MUC1 at 98°C for 20 min before inhibiting endogenous peroxidase activity for 5 min at room temperature (RT) with Tris-EDTA/EGTA pH=9; ii) sections were incubated with a commercially available MUC1 antibody (clone MRQ17, 1:100; CNT) or anti−EGFR monoclonal antibody (clone EP22, 1:50; CNT) for 20 min at RT; iii) an enhanced labelled polymer system (Power Vison) with 3’,3−diaminobenzidine (DAB) was used; and iv) sections were counterstained with hematoxylin. Slides were dehydrated and placed on coverslips.

Evaluation of immunohistochemical staining. Digital images of IHC−stained TMA slides were obtained at x20 magnification using a whole-slide scanner (PRECICE 500 slide scanner; UNIC). Tumor regions on slides were annotated. For each case, total EGFR or MUC1 immunohistochemical staining was evaluated under light microscopy. The staining results were calculated as the histochemistry score (H-score), which was determined by adding the products of the three staining intensity values (weak (1+), moderate (2+), and strong (3+) membranous staining) and their respective cell percentages in the slide.

### Bulk RNA sequencing of PDX samples

RNA of PDX samples was extracted using the Qiagen AllPrep DNA/RNA kit, followed by the library construction using TruSeq RNA sample preparation kit (Illumina), and pair-end sequencing on HiSeq 2000. Quality control of RNA sequencing data was conducted using FastQC (26) and MultiQC (27). Adapter trimming, as well as the removal of low-quality reads (quality score < 30) and short reads (< 30 bp), was performed using Trim Galore (28). Then, the reads were aligned to the Homo sapiens genome (Human GRCh38) using Hisat2 (29) with default parameters. SAM files converted to BAM file using Samtools (30) and gene raw read counts were subsequently extracted from BAM files using featureCounts (31).

### Calculation of RNA cut-off values from IHC and RNA sequencing data of PDX samples

Using the paired RNA sequencing data and IHC scores from the same PDX samples, we aimed to determine the transcriptomic cut-off values for EGFR and MUC1 that would classify samples with positive or negative staining in IHC. Receiver operating characteristic (ROC) curves were drawn using the `roc` function of the R package `pROC` (32), based on the EGFR or MUC1 H-scores from IHC and the log2(TPM+1) values from RNA sequencing of the same PDX samples. The area under the ROC curve (AUC) was calculated, and the best threshold was set as the cut-off.

### TCGA data processing

The TCGA-LUAD and TCGA-COAD datasets were downloaded via the TCGAbiolink package (33). For transcriptomic TPM expression matrix, the data category ‘Transcriptome Profiling’, data type ‘Gene Expression Quantification’, and workflow types “STAR - Counts” and ‘tpm_unstrand’ were selected. TPM value was converted into log2(TPM+1). For clinical information, the data category ‘Clinical’, data type ‘Clinical Supplement’, and data format “BCR XML” were chosen. Additionally, phenotype files of TCGA-LUAD and TCGA-COAD were downloaded from the UCSC Xena web (34) to provide complementary clinical information. For patients with multiple samples, only the tumor sample (sample ID from 0-9) with the latest plate value was retained according to the TCGA barcode guideline. The log-rank test was used to compare Kaplan-Meier survival curves between groups, utilizing the R package `survival` (35). Univariate and multivariate Cox proportional hazards models were applied to evaluate the hazard ratio, using the R package `survminer` (36).

### Statistical analysis

The prognosis of LUAD and CRC patients were estimated through Kaplan-Meier survival curves. The criterion for statistical significance was p < 0.05 in all evaluations unless otherwise indicated.

## Results

### TAA screening based on single-cell meta-atlas

**Figure 1:**
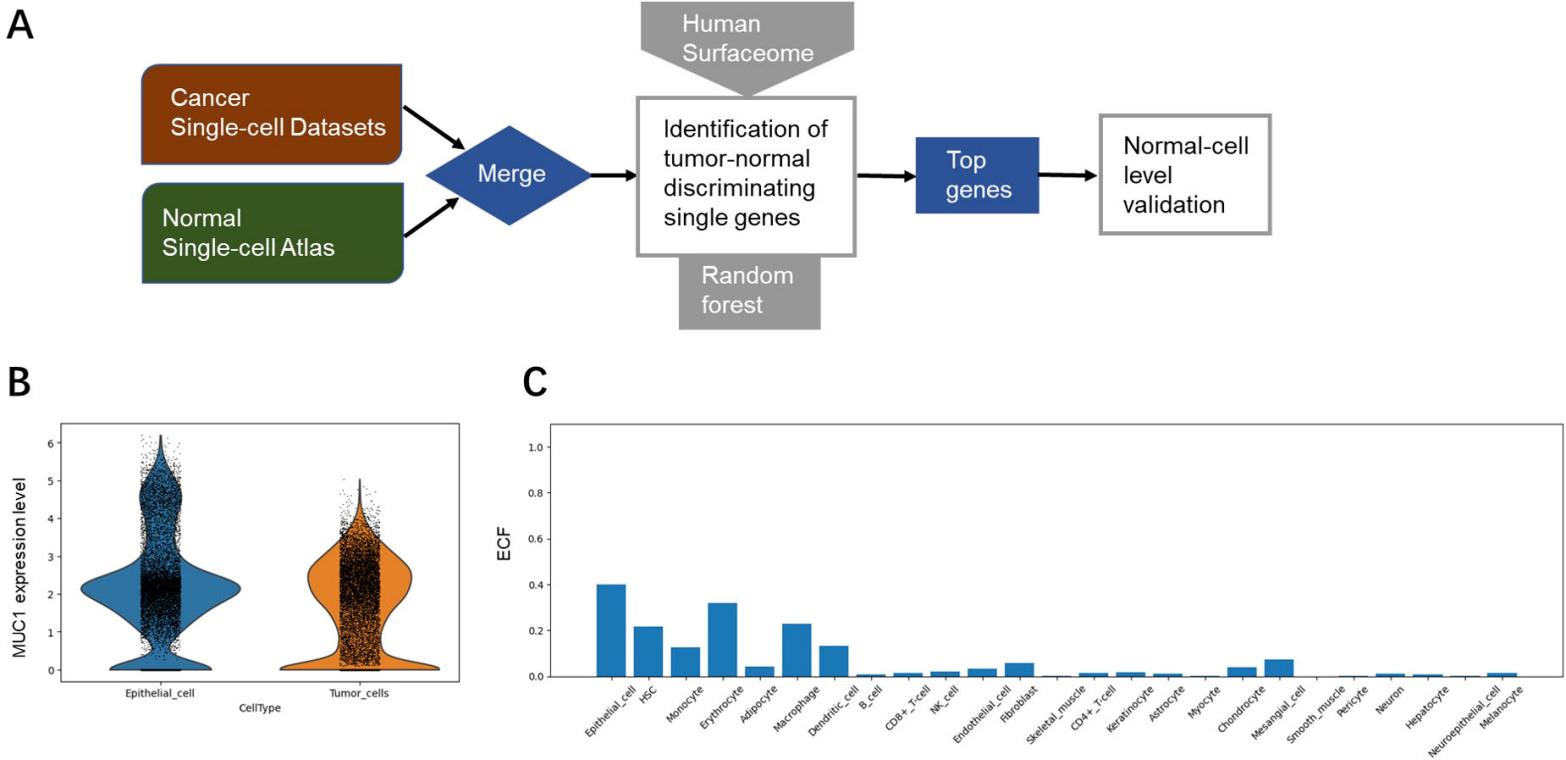
Identification of MUC1 as a potential TAA target. **(A)** Overview of TAA discovery pipeline based on single-cell transcriptomic analysis. **(B)** Expression levels of MUC1 in normal cells (epithelial cell as an example) and tumor cells. **(C)** Expression levels (evaluated by ECF) of MUC1 in various types of normal cells.

To identify TAA targets, we constructed a tumor-normal single cell meta-atlas via integrating multiple single-cell transcriptome datasets of primary lung tumors and normal lung tissues (10–13) for analysis (**Figure 1A**). The ECF of each gene was compared with the average expression level for tumor and normal cell populations, and the genes that contributed most to distinguishing between individual malignant and normal tissues were selected by random forest analysis. Feature importance (FI) was gauged through the metric of ‘mean decrease in accuracy,’ a measure that reflected the model’s proficiency in aligning the surfaceome expression profiles with the initial labels of tumor or normal as annotated by our meta-atlas. The top 100 genes with the highest FI were selected for detailed literature review, with various aspects taken into consideration, such as drug developmental status, signaling pathway, gene expression and mutations. Among them MUC1 was finally selected with low expression in various normal cell types for subsequent evaluation (**Figures 1B, C**).

### Single-cell analysis confirmed the co-expression of EGFR and MUC1 in tumor cells

**Figure 2:**
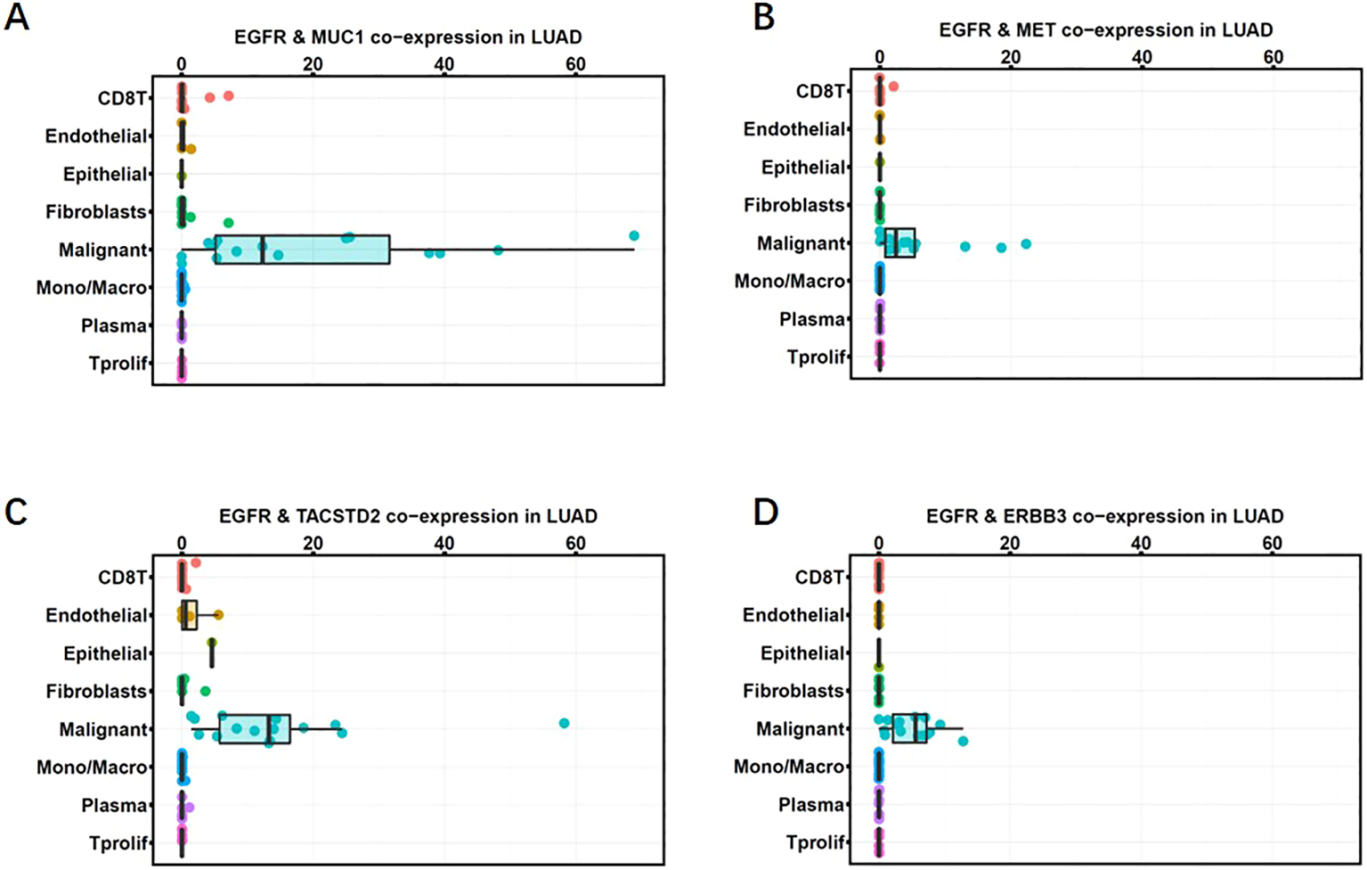
Co-expression of MUC1 and EGFR in LUAD single-cell RNA sequencing data (GSE148071). Co-expression percentages of EGFR with MUC1 **(A)**, MET **(B)**, TROP2 **(C)**, HER3 **(D)** in different cell clusters in the tumor microenvironment are shown as box plots. Gene expression was quantified as log2(TPM+1). Each dot represents one patient. The horizontal line in the box plot denotes the median, and the box denotes the 25th to 75th quantiles of the data.

We performed single-cell analysis based on data from an independent study (14) to validate the co-expression of EGFR and MUC1 in cancer cells, as well as in other cells of the tumor microenvironment. We used the cut-off of raw count values > 0 to define co-expression of genes at single-cell transcriptomic level. Co-expression of EGFR and MUC1 in malignant cells and stromal cells, including fibroblasts, endothelial cells, normal epithelial cells and immune cells (conventional CD4 T cells, CD8 T cells, exhausted CD8+ T cells, proliferating T cells, NK cells, monocytes or macrophages) was estimated. Co-expression of EGFR and MUC1 in stromal cells was lower than 10% (mostly under 5%) in any patient (**Figure 2**; **Supplementary Table 3**). By contrast, 44% of LUAD patients had more than 10% EGFR and MUC1 co-expression in malignant cells, which was significantly higher than the co-expression percentages in other cell populations (P-value = 2.088e-10). The co-expression of EGFR and MUC1 was higher in tumor cells than EGFR combined with targets under active drug development for lung cancer such as MET, HER3 (ERBB3) or Trop2 (TACSTD2) (**Figure 2**), suggesting the potential safety advantage of developing therapies targeting EGFR and MUC1.

### IHC analysis of MUC1 and EGFR expression in normal tissues and tumor samples

**Figure 3:**
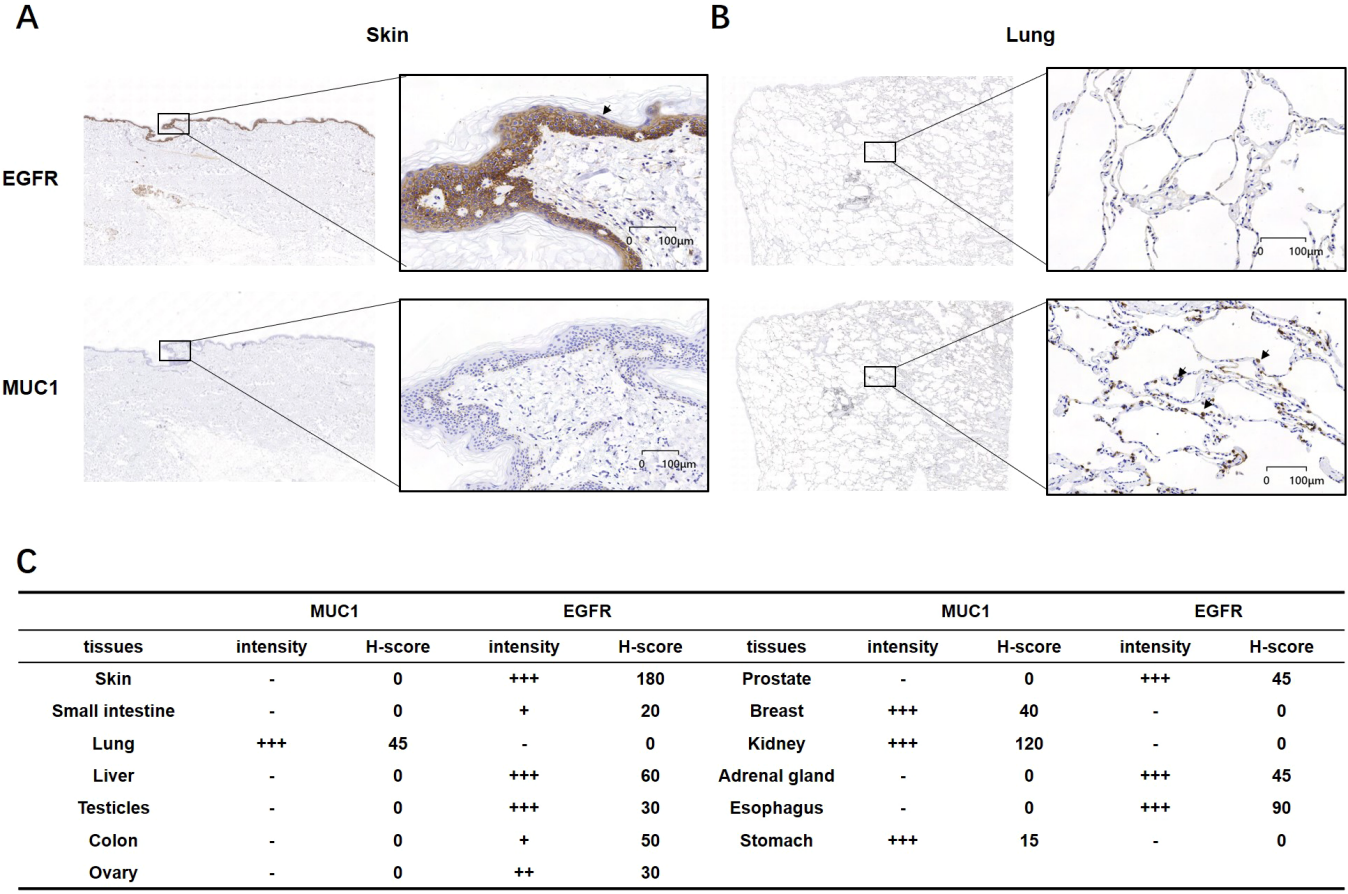
IHC analysis of MUC1 and EGFR in normal human tissues. **(A, B)** Representative images of EGFR and MUC1 showing no co-expression in normal skin and lung tissues. **(C)** Intensity and H-score of EGFR and MUC1 in various normal tissues. The symbols represent the staining intensity: -, negative; +, weak; ++, moderate; +++, strong.

To examine the protein levels of MUC1 and EGFR in normal tissues, we conducted IHC on a panel of tissue samples from different anatomic sites (**Table 1**). IHC method for EGFR and MUC1 was established and optimized by screening the most specific primary antibody and testing appropriate antibody concentration (**Supplementary Figure 1**). EGFR showed positive staining in skin, liver, testicle, prostate, adrenal gland and esophagus, while MUC1 displayed more restricted staining in lung, breast, kidney and stomach (**Figure 3**). Importantly, the data suggested that these two targets were not co-existed in any normal tissues, which is crucial to minimize potential on-target, off-tumor toxicity.

**Figure 4:**
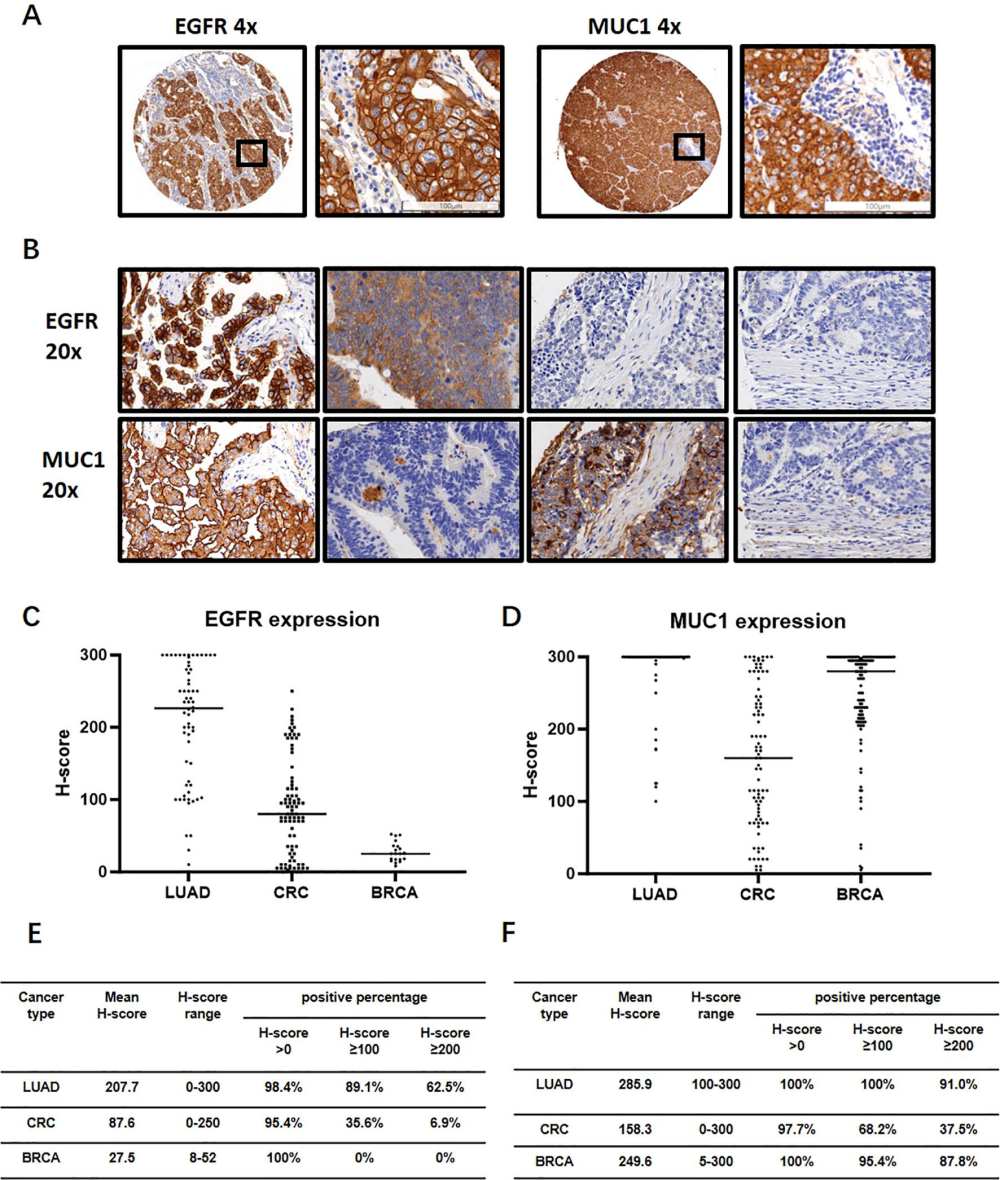
IHC analysis of EGFR and MUC1 in LUAD, CRC and breast invasive carcinoma (BRCA) clinical samples. **(A)** Representative images of EGFR and MUC1 staining profiles. The image in the black box on the right is a magnification of the black box on the left to visualize the tumor cells. **(B)** Representative images showing co-expression, differential expression and non-expression of EGFR and MUC1 in consecutive sections of LUAD and CRC samples. **(C, D)** Expression levels of EGFR and MUC1 quantified by H-score in LUAD, CRC and BRCA. **(E, F)** Percentages of samples with different cut-offs of H-score for EGFR **(E)** and MUC1 **(F)**.

As aberrant expression of EGFR and MUC1 has been reported in various epithelial tumors (37–39), we carried out IHC experiments on LUAD, CRC and breast cancer tissues (**Table 2**). EGFR generally showed the membranous staining pattern, while MUC1 exhibited both membranous and cytoplasmic patterns, consistent with previous reports (40–42) (**Figure 4A**). Of the 64 clinical LUAD samples analyzed, EGFR protein expression was observed in 63 (98.4%) cases, and MUC1 were stained positive in all of the samples. Among CRC clinical samples, 79 (95.2%) and 81 (97.6%) were positive for EGFR and MUC1, respectively. As EGFR and MUC1 were stained in consecutive sections with a thickness of 4 μm, which was smaller than the averaged diameter of tumor cells (7-20 μm in most cases), we could observe if they were co-expressed in the same tumor cells (**Figure 4B**). Roughly, EGFR and MUC1 were detected on the same tumor cells in 63 (98.4%) LUAD and 76 (91.6%) CRC samples, which included samples with at least 1% of tumor cells stained positive with both EGFR and MUC1 regardless of staining intensity.

The H-score, which was the sum of the staining intensities multiplied by their corresponding cell percentages, averaged 207.7 (EGFR) and 285.9 (MUC1) in LUAD and 87.6 (EGFR) and 158.3 (MUC1) in CRC (**Figures 4C–F**). Using H-score ≥100 and H-score ≥200 as the cut-off values for medium and high expression of each protein, medium levels of EGFR and MUC1 were found in 54 (84.3%) LUAD and 19 (22.9%) CRC samples (**Supplementary Figure 2**). Furthermore, 60.9% of LUAD and 3.6% of CRC samples showed high levels of EGFR and MUC1 (**Supplementary Figure 2**). However, the staining intensity and percentage of EGFR were much weaker in breast cancer samples which were therefore not used for the following analysis (**Figures 4C, E**).

Taken together, IHC analysis demonstrated that EGFR and MUC1 were present at medium to high levels in the majority of LUAD and CRC samples, and in line with single-cell analysis, they tended to be co-expressed on tumor cells. Moreover, they did not show high expression or co-existence patterns in normal tissues.

### EGFR-MUC1 as dual-TAA targets could potentially cover a large population of NSCLC patients

**Figure 5:**
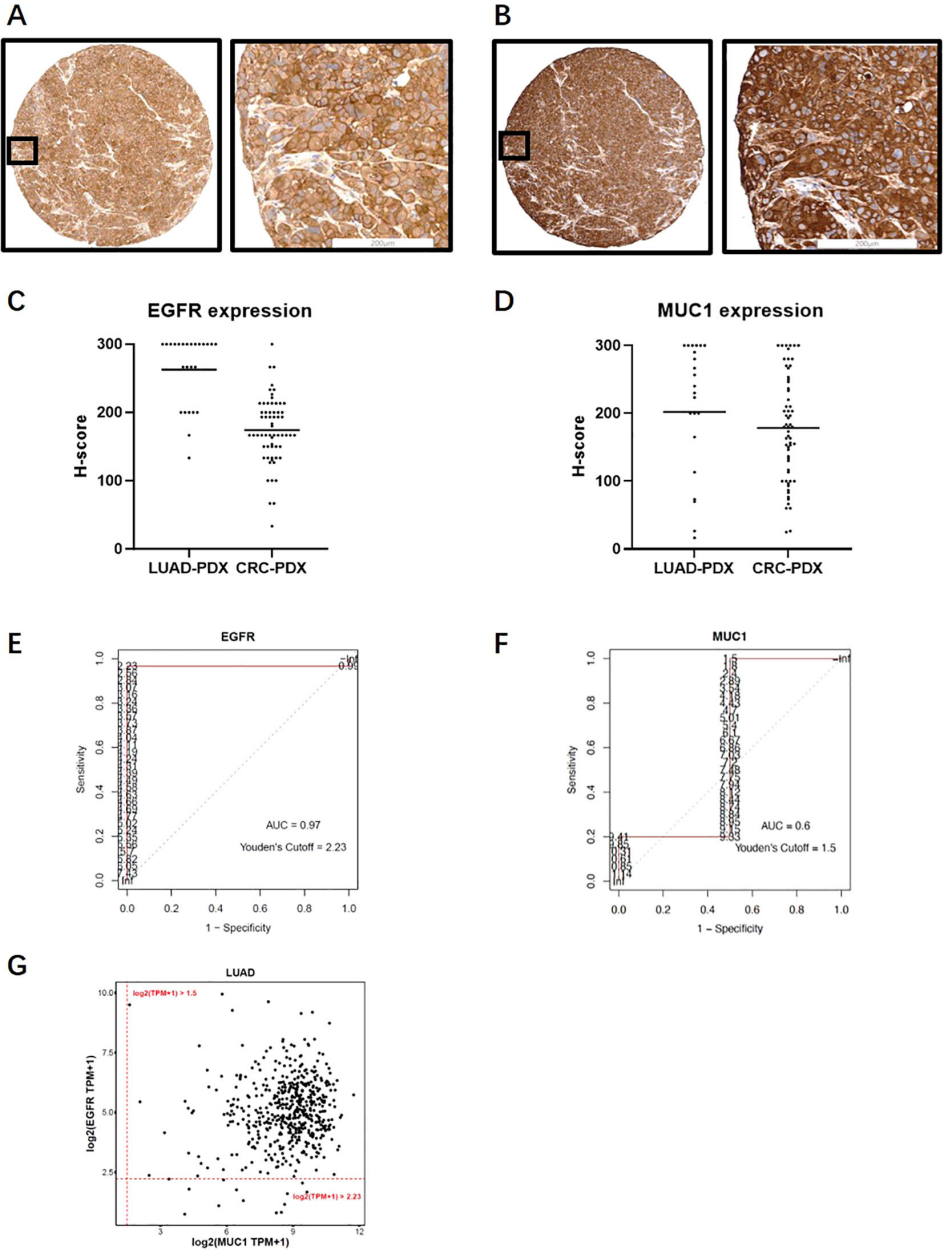
Expression analysis of EGFR and MUC1 in PDX samples and TCGA database. **(A, B)** Representative images of EGFR **(A)** and MUC1 **(B)** staining profiles in PDX samples. **(C, D)** EGFR and MUC1 expression levels quantified by H-score in LUAD and CRC PDX samples. **(E, F)** ROC curves for EGFR **(E)** and MUC1 **(F)** in LUAD PDX samples which were drawn using H-score and RNA log2(TPM+1) data from the same PDX sample. AUCs and log2(TPM+1) threshold values corresponding to positive protein staining by Youden’s cut-off are also illustrated. **(G)** Estimated percentage of EGFR and MUC1 expression in LUAD patients in the TCGA database.

To estimate the size of patient population with EGFR and MUC1 expression using TCGA database, we calculated the cut-off values of RNA expression corresponding to the positivity of EGFR and MUC1 protein levels. We used PDX samples due to their similarity to primary tumors, and obtained RNA sequencing and IHC staining results of the same PDX samples. In 24 and 62 PDX samples of LUAD and CRC respectively, the staining patterns of EGFR and MUC1 were similar to those in primary clinical samples (**Figures 5A, B**). 100% LUAD samples (mean H-score=262) and CRC samples (mean H-score=174) were positive for EGFR, while 95.8% LUAD samples (mean H-score=202) and 100% CRC samples (mean H-score=178) were positive for MUC1 (**Figures 5C, D**). The cut-off log2(TPM+1) values were determined to predict IHC positivity through ROC curve analysis, in which a larger AUC indicated a better predictive performance (a higher consistency between TPM and H-score). **Figures 5E, F** showed the ROC curves and the AUC values for LUAD PDX samples, illustrating a high AUC value (0.97 by H-score >0) for EGFR, a medium AUC value (0.6 by H-score >0) for MUC1 in LUAD, and a low AUC value for both EGFR and MUC1 in CRC (data not shown). Therefore, we proceeded with analyses of EGFR and MUC1 in LUAD.

We estimated the optimal threshold value of RNA expression referring to protein positivity (H-score >0) by the roc function in the R package “pROC” as the cut-off (43). The log2(TPM + 1) cutoff points of 2.23 for EGFR and 1.5 for MUC1 (**Figures 5E, F**) were applied in the TCGA database to define positivity. Consistent with our above results, 504 of 517 (97.5%) LUAD samples were positive for EGFR, and MUC1 protein expression was positive in 517 cases (100%). Thus, the proportion of tumor samples positive with EGFR and MUC1 was estimated to be 97.5% (**Figure 5G**), which indicated that a large population of LUAD patients could potentially benefit from EGFR-MUC1 dual-targeting therapies.

### EGFR and MUC1 corelated with poor prognosis of LUAD and CRC patients

**Figure 6:**
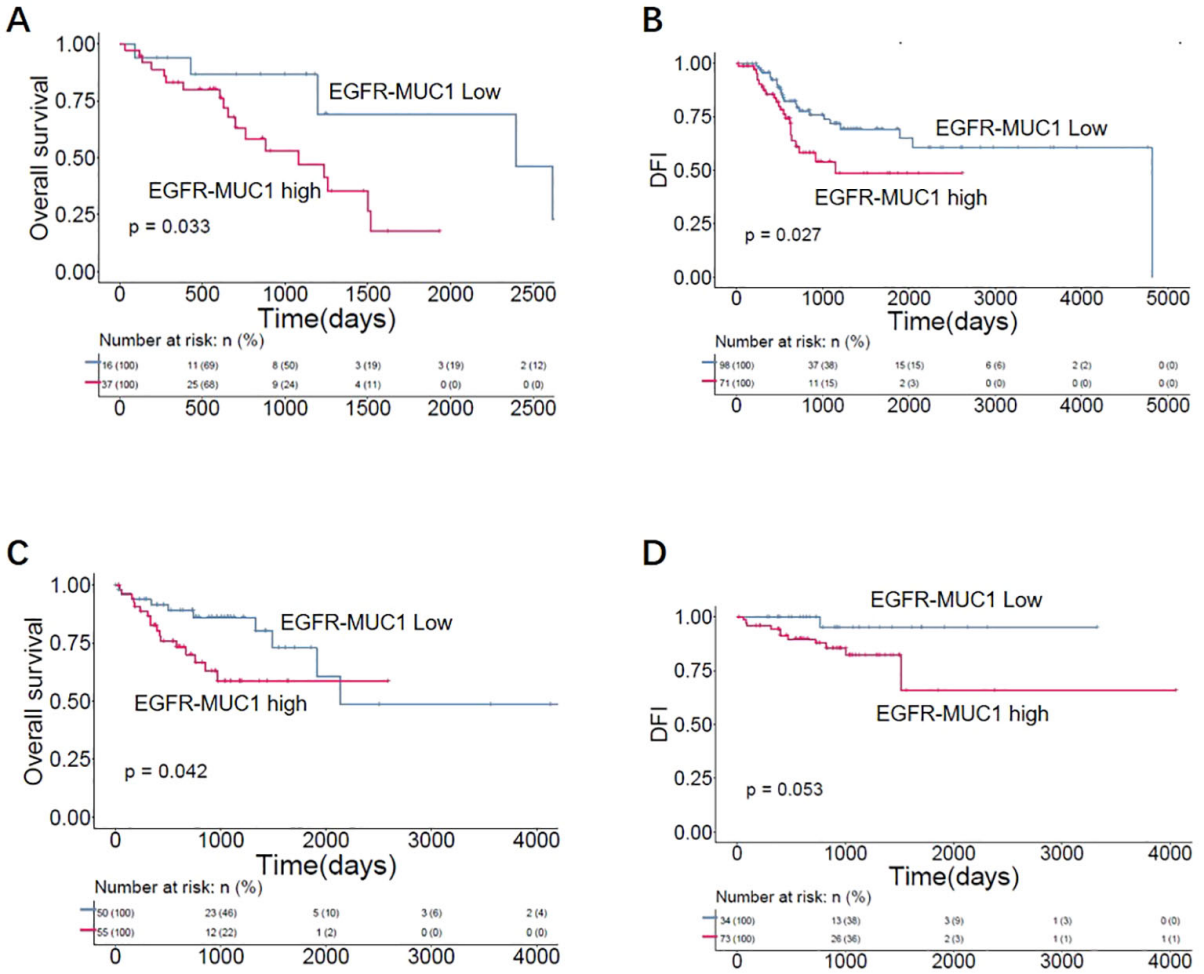
Correlation of EGFR-MUC1 expression with prognosis of LUAD and CRC patients. Survival curves showing the association between EGFR-MUC1 expression and prognosis of LUAD **(A, B)** and CRC patients **(C, D)**.

To further evaluate the clinical significance of EGFR and MUC1 expression, we analyzed the correlation between EGFR and MUC1 expression and the prognosis of LUAD and CRC patients through Kaplan-Meier survival curves. To predict the effect of EGFR and MUC1 on the survival of individuals who might need biological treatments, patients with advanced tumor stages (TNM tumor stage III/IV) from TCGA database were selected for the survival analysis. In LUAD patients, although there was no significant difference, we observed that high expression of EGFR or MUC1 alone was corelated with reduced overall survival (OS) and disease-free interval (DFI) (**Supplementary Figures 3A–D**). Furthermore, LUAD patients with high expression of both EGFR and MUC1 had significantly worse prognosis than other patients (**Figures 6A, B**, p-value=0.033 for OS, p-value=0.027 for DFI). In CRC patients, prognosis of patients with high EGFR or MUC1 expression alone was slightly worse than low expression (**Supplementary Figures 3E–H**), while the prognosis of patients with high expression of both EGFR and MUC1 was significantly worse than that of patients with low expression levels of both targets (**Figures 6C, D**, p-value=0.045 for OS, p-value=0.053 for DFI). These results suggested high expression of EGFR-MUC1 might serve as a prognostic factor for LUAD and CRC patients, and development of dual- targeting therapies might provide effective treatment options for patients with late stage LUAD or CRC.

## Discussion

The EGFR-dependent pathway has an important role in epithelial cancer biology, which has led to the development of cetuximab and necitumumab. However, severe skin toxicity, most likely due to the expression of EGFR in normal epithelium, hinders the widespread use of these drugs (7). To increase tumor selectivity and reduce tumor escape, dual-targeting therapies using antibodies, antibody-drug conjugates (ADCs) or chimeric antigen receptor T cells have emerged (44, 45), as targeting two TAAs simultaneously might better distinguish tumor cells from normal cells.

In this work, we screened TAA targets using a single-cell analysis pipeline, and found a potential target MUC1 with high expression in tumor cells and relatively low or undetectable expression in normal tissues. MUC1 is a glycoprotein involved in the proliferation, metabolism, metastasis and invasion of multiple tumor types (46–49), and overexpressed in various epithelial cancers with aberrant glycosylation (50). As MUC1 protein was confined to the apical surface of epithelial cells under normal physiological conditions, it was distributed all over the cell surface and within the cytoplasm in tumor cells, which might improve the safety of drugs targeting MUC1 (47). Thus, MUC1 presented unique properties in cancer cells, including the aberrant glycosylation pattern, loss of polarity and overexpression, making it an attractive TAA target (47). However, the clinical efficacy of monotherapy targeting MUC1 was much lower than expected. The glyco-engineered humanized monoclonal antibody PankoMab-GEX showed no difference from placebo for the primary endpoint of progression-free survival (51). One possible reason could be that these anti-MUC1 antibodies were developed to target the N-terminal subunit (MUC1-N) which was usually shed from the cell surface and released into the peripheral blood. The detached MUC1-N attached to antibodies and prevented them from binding to surface MUC1 (52).

Several studies have demonstrated that MUC1 drove EGFR expression and signaling in different cellular contexts (53–56). Through its C-terminus, MUC1 stimulated EGFR promoter activation, thereby increasing EGF-dependent signaling, spheroid survival and cellular proliferation (55). Furthermore, knockout of MUC1 in tumor cells resulted in higher sensitivity to EGFR inhibitors, and activated EGFR stimulated MUC1 expression in human uterine and pancreatic cancer cell lines (55). Therefore, it is predicted that EGFR-MUC1 dual-targeting therapies could improve the response of tumor cells, and analysis of EGFR and MUC1 co-expression patterns in clinical samples are essential to provide a solid rationale for drug development.

We investigated the expression patterns of EGFR and MUC1 in patients with LUAD and CRC by IHC, and observed high expression of EGFR and MUC1 in tumor tissues. In contrast, there was lower expression of both proteins in various normal tissues. In line with previous studies, co-expression of EGFR and MUC1 in the same tumor cells was found in most cases, including 63 (98.4%) LUAD and 76 (91.6%) CRC samples. Using the RNA cut-off values for EGFR and MUC1 positivity, we estimated that 97.5% of LUAD patients expressed EGFR and MUC1 based on the 517 cases’ data from TCGA, which suggested that EGFR and MUC1 expression was prevalent in LUAD patients. Furthermore, high expression of EGFR and MUC1 was prognostic for poor survival of LUAD and CRC patients.

There are some limitations in our study. Firstly, as we showed the expression patterns of EGFR and MUC1 in a panel of normal tissues and various tumor tissues, the sample size was still limited to achieve a thorough comparison between tumor and normal tissues. A larger collection of normal samples and tumor samples with paired tumor-adjacent tissues need to be examined to better elucidate the potential toxicity of targeting EGFR and MUC1. Secondly, we did not show experimental data to confirm the efficacy and safety of dual-targeting strategy in this study, while other companies have reported their preclinical data on bispecific ADCs targeting EGFR and MUC1 recently, which suggested the great therapeutic potential of dual-targeting drugs for cancers co-expressing EGFR and MUC1, as well as EGFR- or MUC1- expressing tumors (57, 58).

In summary, through a generalizable pipeline for screening TAAs by single-cell transcriptomic and IHC analysis, MUC1 was selected as a candidate target to combine with EGFR, as to increase the tumor specificity over normal cells. We demonstrated that EGFR and MUC1 were co-expressed on tumor cells in the majority of LUAD and CRC clinical samples. High expression of EGFR and MUC1 was corelated with unfavorable prognosis of LUAD and CRC patients. Given that EGFR and MUC1 expression was present in a large patient population, our work has shed light on the prospects of developing EGFR-MUC1 dual-targeting therapies, such as bispecific antibodies and ADCs, which might broaden the tumor selectivity while reducing the side-effects on normal organs.

## Data Availability

The public datasets used in this study are available in the TCGA database (https://www.cancer.gov/tcga), ArrayExpress database (https://www.ebi.ac.uk/biostudies/arrayexpress), and Tissue Stability Cell Atlas (https://www.tissuestabilitycellatlas.org/). The details can be found in the article and its **Supplementary Materials**.

## Supplementary material

Supplementary Figure 1 Establishment of IHC method and evaluation of staining intensity. Representative examples of IHC method optimization **(A)** and positive samples with different staining intensities **(B)**. **(A)** An optimized antibody concentration of 0.5 μg/mL was selected considering MUC1 staining sensitivity and specificity in tonsil tissue. **(B)** Membrane staining was scored as follows: 0 for no staining visible at a magnification of x400; 1+ for light staining visible at a magnification of x400; 2+ for intermediate staining visible at a magnification of x400; and 3+ for dark staining of the linear membrane visible at a magnification of x100.

Supplementary Figure 2 Percentages of EGFR and MUC1 expression in LUAD **(A)** and CRC **(B)** samples according to different H-score cut-offs (H-score>0, H-score ≥100, H-score ≥200).

Supplementary Figure 3 Correlation of EGFR or MUC1 expression with prognosis in LUAD and CRC patients. **(A, B)** Survival curves showing the association between EGFR expression and OS rate **(A)** or DFI **(B)** of LUAD patients. **(C, D)** Survival curves showing the association between MUC1 expression and OS rate **(C)** or DFI **(D)** of LUAD patients. **(E, F)** Survival curves showing the association between EGFR expression and OS rate **(E)** or DFI **(F)** of CRC patients. **(G, H)** Survival curves showing the association between MUC1 expression and OS rate **(G)** or DFI **(H)** of CRC patients.
