# Supplementary figures and images for: EGFR and MUC1 as dual-TAA drug targets for lung cancer and colorectal cancer
Source: Front Oncol. 2024 Nov 27;14:1433033. doi: 10.3389/fonc.2024.1433033 (PMC11631732; doi:10.3389/fonc.2024.1433033)

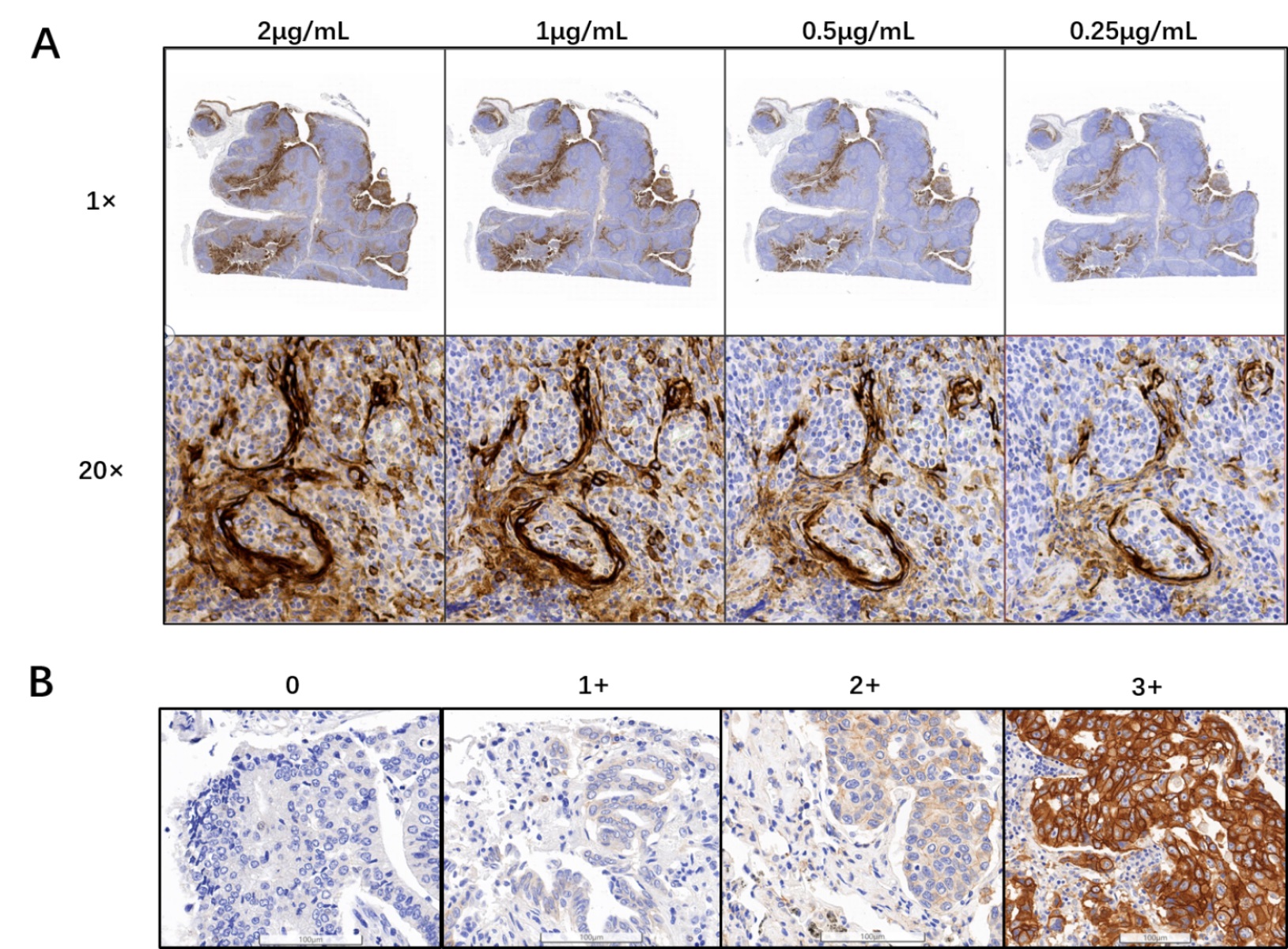

Supplement: Supplementary Figure 1 — Establishment of IHC method and evaluation of staining intensity. Representative examples of IHC method optimization (A) and positive samples with different staining intensities (B). (A) An optimized antibody concentration of 0.5 μg/mL was selected considering MUC1 staining sensitivity and specificity in tonsil tissue. (B) Membrane staining was scored as follows: 0 for no staining visible at a magnification of x400; 1+ for light staining visible at a magnification of x400; 2+ for intermediate staining visible at a magnification of x400; and 3+ for dark staining of the linear membrane visible at a magnification of x100. [file Image1.jpg]

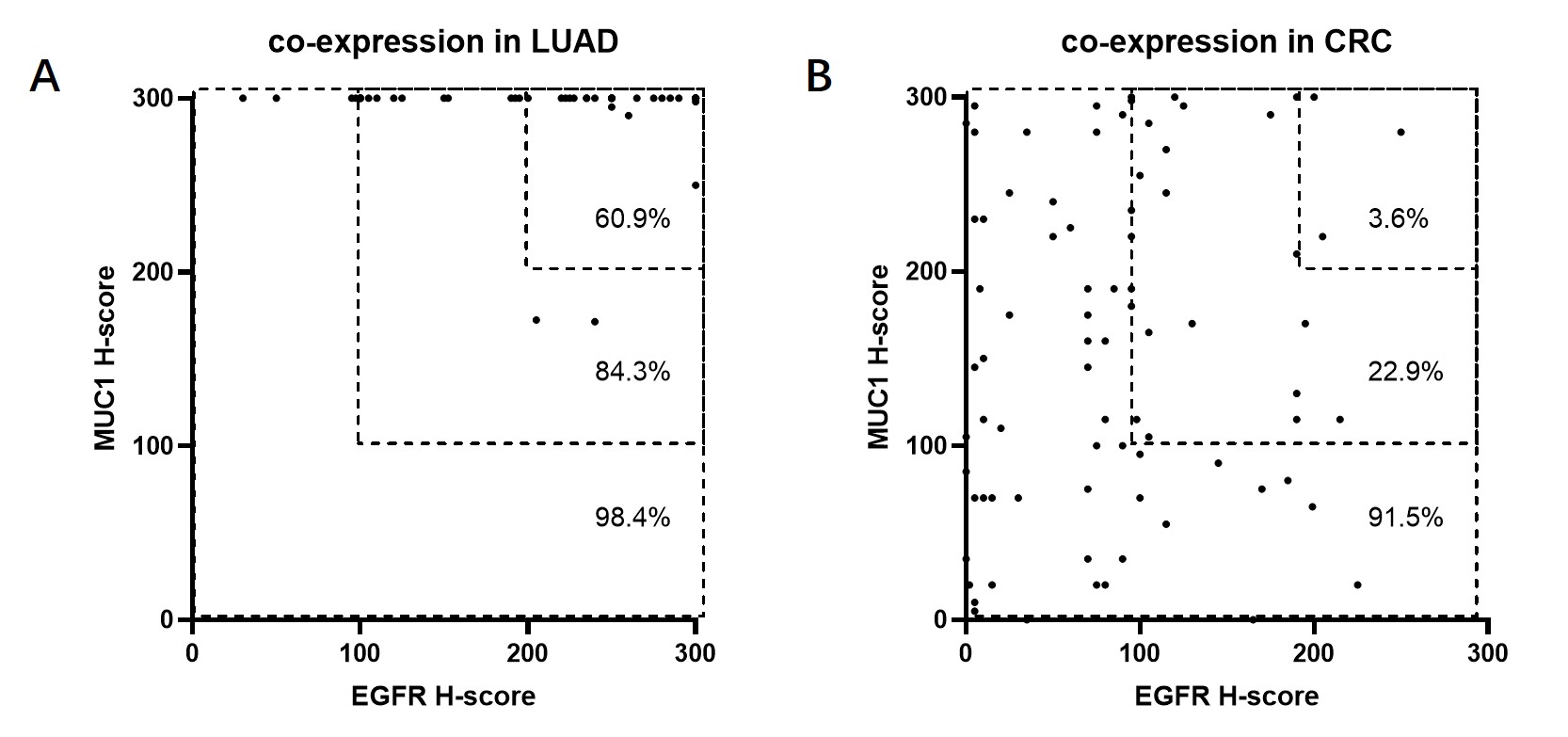

Supplement: Supplementary Figure 2 — Percentages of EGFR and MUC1 expression in LUAD (A) and CRC (B) samples according to different H-score cut-offs (H-score>0, H-score ≥100, H-score ≥200). [file Image2.jpg]

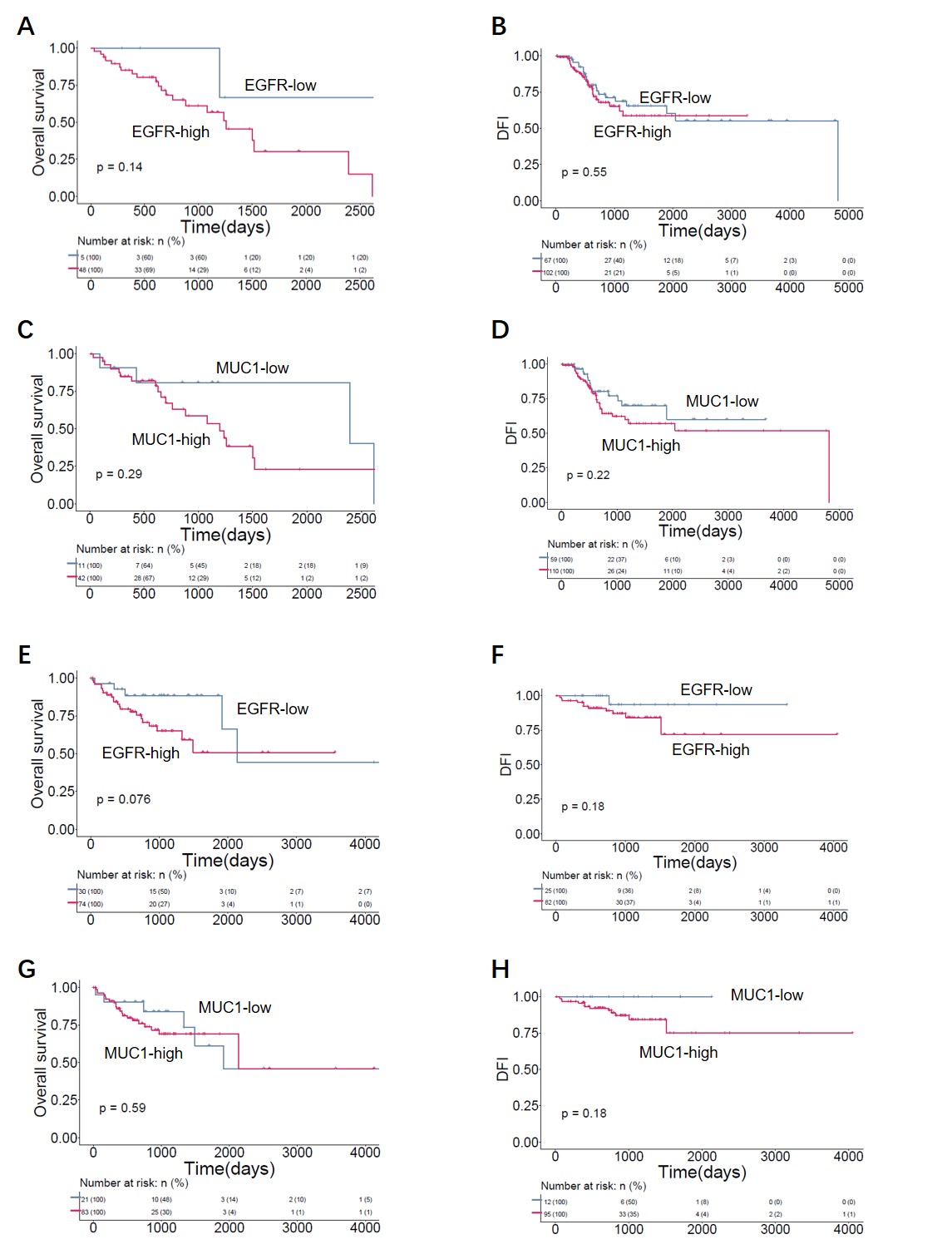

Supplement: Supplementary Figure 3 — Correlation of EGFR or MUC1 expression with prognosis in LUAD and CRC patients. (A, B) Survival curves showing the association between EGFR expression and OS rate (A) or DFI (B) of LUAD patients. (C, D) Survival curves showing the association between MUC1 expression and OS rate (C) or DFI (D) of LUAD patients. (E, F) Survival curves showing the association between EGFR expression and OS rate (E) or DFI (F) of CRC patients. (G, H) Survival curves showing the association between MUC1 expression and OS rate (G) or DFI (H) of CRC patients. [file Image3.jpg]
